# Supplementary material for: Knowledge of Sexuality and Contraception in Students at a Spanish University: A Descriptive Study
Source: Healthcare (Basel). 2022 Sep 5;10(9):1695. doi: 10.3390/healthcare10091695 (PMC9498656; doi:10.3390/healthcare10091695)
Supplement: Supplementary file 1 [file healthcare-10-01695-s001.zip › Suplementary file S1.pdf]

Estimado participante,

El cuestionario que a continuación vas a rellenar es anónimo y sus respuestas serán confidenciales. El objetivo de esta investigación es conocer el nivel de conocimiento que tenéis sobre prevención del embarazo no deseado.

A continuación te exponemos algunas cuestiones, a las que rogamos que respondas con la mayor sinceridad. Queremos recordarte que no hay respuestas "buenas" y "malas", responde lo que pienses que es más correcto.

Por último queremos informarte que el resultado de este cuestionario no influirá de ninguna manera en la calificación de ninguna de las asignaturas que actualmente cursas.

Atentamente:
Los investigadores.

Muchas gracias por tu colaboración.

Dominio 1.

1) Indica tu sexo:

- Hombre (1) [ ]
- Mujer (2) [ ]

2) Indica tu edad:

- …………..

3) El colegio donde estudia es:

- Público (1) [ ]
- Privado (2) [ ]

4) Señale de las siguientes opciones, cual es la fuente de información que usa con mayor frecuencia para obtener información sobre sexualidad:

- Internet. (1) [ ]
- Televisión. (2) [ ]
- Amigos. (3) [ ]
- Charlas sobre salud reproductiva. (4) [ ]
- Padres. (5). [ ]
- Otros (Especificar):………………. (6) [ ].

5) ¿A través de qué fuente querrías recibir la información sexual?

- Internet. (1) [ ]
- Televisión. (2) [ ]
- Folletos entregados en su escuela. (3) [ ]
- Otros (Indicar cuales) (4): [ ]

6) ¿Cómo consideras tu formación sobre sexualidad?

- Buena (1). [ ]
- Regular (2). [ ]
- Mala. (3). [ ]

7) Si tienes una duda puntual sobre sexualidad, ¿a dónde acudirías para resolverla?

- Chat con amigos. (1) [ ]
- Páginas web. (2) [ ]
- A tus padres. (3) [ ]
- Otros. (4)(Indicar cual): [ ]

8) ¿Has tenido relaciones sexuales completas?

- Si. (1) [ ]
- No. (2) [ ] Continua con la pregunta 18

9) ¿Qué edad tenías cuando tuviste tu primera relación?

- …………..

10) ¿Qué motivo te llevó a tener relaciones sexuales?

- Para sentirme aceptado/a y amado/a. (1) [ ]
- Porque era lo que se esperaba que hiciera. (2) [ ]
- Por placer. (3) [ ]
- Porque amo o amaba a mi pareja. (4) [ ]
- Para mantener a mi novio/a conmigo. (5) [ ]
- Otros. (6) (Indicar cual): [ ]

11) ¿Usasteis algún método anticonceptivo?

- Si (1) [ ]
- No (2) [ ]. Pasar a la pregunta 13

12) ¿Qué método anticonceptivo usasteis?

- Preservativo masculino. (1) [ ]
- Píldora del día después. (2) [ ]
- Preservativo femenino. (3) [ ]
- Otros. (4) (Indicar cual): [ ]

13) ¿Qué método anticonceptivo usarías si la tuvieras?

- Preservativo masculino. (1) [ ]
- Píldora del día después. (2) [ ]
- Preservativo femenino. (3) [ ]
- Otros. (4) (Indicar cual): [ ]

14) No hay riesgo de embarazo al tener relaciones sexuales durante la menstruación.

- Verdadero. [ ]
- Falso. [ ]

15) Después de tener relaciones sexuales no hay cambios físicos que hacen que las demás personas sepan que has tenido relaciones sexuales.

- Verdadero. [ ]
- Falso. [ ]

16) En la primera relación sexual con una mujer virgen, esta se puede quedar embaraza

- Verdadero. [ ]
- Falso. [ ]

17) Lavarse la vagina inmediatamente después de tener relaciones sexuales previene el embarazo.

- Verdadero. [ ]
- Falso. [ ]

18) Si una chica tiene relaciones sexuales pero no llega al orgasmo, no hay riesgo de embarazo.

- Verdadero. [ ]
- Falso. [ ]

19) El preservativo es seguro si se coloca justo antes de correrse.

- Verdadero. [ ]
- Falso. [ ]

20) Usar dos preservativos masculinos a la vez es más seguro, para prevenir un embarazo no deseado.

- Verdadero. [ ]
- Falso. [ ]

21) Un inconveniente para usar los preservativos es que las relaciones sexuales son menos placenteras.

- Verdadero. [ ]
- Falso. [ ]

22) El preservativo masculino es un método seguro para tener relaciones sexuales.

- Verdadero. [ ]
- Falso. [ ]

23) Se puede usar otros lubricantes además del que trae el preservativo, sin inconvenientes.

- Verdadero. [ ]
- Falso. [ ]

24) El “Método del calendario” no es recomendable para evitar un embarazo.

- Verdadero. [ ]
- Falso. [ ]

25) La toma de la píldora anticonceptiva de emergencia puedo demorarla hasta 72 horas sin que pierda efectividad.

- Verdadero. [ ]
- Falso. [ ]

26) La píldora anticonceptiva debe tomarse a la misma hora siempre pero si se olvida y han pasado menos de 12 horas, me puedo la puedo tomar, siempre que la siguiente toma sea a la hora original sin que pierda efectividad anticonceptiva:

- Verdadero. [ ]
- Falso. [ ]

27) La píldora anticonceptiva es efectiva desde el primer día, siempre que se inicie a la misma vez que la menstruación.

- Verdadero. [ ]
- Falso. [ ]

28) Los métodos anticonceptivos hormonales no son adecuados para los adolescentes.

- Verdadero. [ ]
- Falso. [ ]

29) El uso de píldoras anticonceptivas causa que la mujer engorde y le salga acné en la piel.

- Verdadero. [ ]
- Falso. [ ]

Dear participant,

The following questionnaire is anonymous and your answers are confidential. The aim of this research is to know the level of knowledge you have about unwanted pregnancy prevention.

We ask you to answer sincerely to 29 questions. We want to remind you that there are no "good" or "bad" answers, answer whatever you think is most correct.

Finally, we would like to inform you that the result of this questionnaire will not influence, anyway, in the mark of any of the subjects you are currently studying.

Yours sincerely,
The researchers.

Thank you very much for your participation.

1) Indicate your gender:

- Man (1) [ ]
- Woman (2) [ ]

2) Indicate your age:

- …………..

3) The school where you studied was:

- Public (1) [ ]
- Private (2) [ ]

4) Which of the following sources of information do you use most often to obtain information about sexuality and contraception?

- Internet. (1) [ ]
- Television. (2) [ ]
- Friends. (3) [ ]
- Lectures about reproductive health. (4) [ ]
- Parents. (5). [ ]
- Other (please specify): .... (6) [ ].

5) By what source would you like to receive the sexual information?

- Internet. (1) [ ]
- Television. (2) [ ]
- Brochures delivered for your educational institution. (3) [ ]
- Other (please specify): .... (4) [ ]

6) How do you consider your training on sexuality and contraception?

- Good (1). [ ]
- Regular (2). [ ]
- Bad. (3). [ ]

7) If you have a specific question about sexuality, where would you go to solve it?

- Chat with friends. (1) [ ]
- Web pages. (2) [ ]
- To your parents. (3) [ ]
- Others. (please specify): .... (4) [ ]

8) Have you had complete sexual intercourse?

- Yes. (1) [ ]
- No. (2) [ ] Continue with question 13

9) How old were you when you had your first intercourse?

- …………..

10) What led you to have sex?

- To feel me accepted and loved. (1) [ ]
- Because that's what it was expected I do. (2) [ ]
- For pleasure. (3) [ ]
- Because I love or loved my partner. (4) [ ]
- To keep my boyfriend/girlfriend with me. (5) [ ]
- Others (Please Specify) .... (6) [ ]

11) Did you use any contraceptive method during your first sexual intercourse?

- Yes (1) [ ]
- No (2) [ ]. Continue to question 13

12) Which contraceptive method did you use?

- Male condom. (1) [ ]
- Emergency contraceptive Pill. (2) [ ]
- Contraceptive Pills. (3) [ ]
- Others. (Please Specify): .... (4) [ ]

13) What contraceptive method would you use if you had a partner or in future sexual relations?

- Male condom. (1) [ ]
- Emergency contraceptive Pill. (2) [ ]
- Contraceptive Pills. (3) [ ]
- Others. (Please Specify): .... (4) [ ]

14) There isn’t any risk of pregnancy if you have sexual intercourse during menstruation.

- True. [ ]
- False. [ ]

15) At the first sexual intercourse with a virgin woman, she may become pregnant.

- True. [ ]
- False. [ ]

16) The 'Calendar Rhythm Method' is not recommended to prevent a pregnancy.

- True. [ ]
- False. [ ]

17) Abstinence is the only contraceptive method with a 100% success rate.

- True. [ ]
- False. [ ]

18) The "pulling out" method of contraception is a very effective contraceptive to prevent a pregnancy.

- True. [ ]
- False. [ ]

19) The responsibility for the use of any contraceptive method is shared by both partners.

- True. [ ]
- False. [ ]

20) The condom is safe if you put on just before ejaculation.

- True. [ ]
- False. [ ]

21) Using two male condoms at the same time is safer to prevent unwanted pregnancy.

- True. [ ]
- False. [ ]

22) The male condom is a safe method for having sex.

- True. [ ]
- False. [ ]

23) It can be used others Lubricants than those provided by the male condom without risk.

- True. [ ]
- False. [ ]

24) The male condom can be used several times.

- True. [ ]
- False. [ ]

25) I can delay taking the emergency contraceptive pill for up to 72 hours without losing its effectiveness.

- True. [ ]
- False. [ ]

26) The contraceptive pills should always be taken at the same hour, but if it is a forgotten and less than 12 hours have passed, I can take it, and continue with the initial timing without losing contraceptive effectiveness:

- True. [ ]
- False. [ ]

27) The contraceptive pills are effective from day one, as long as you start to dose it at the same time as menstruation.

- True. [ ]
- False. [ ]

28) Hormonal contraceptive methods are not suitable for adolescents.

- True. [ ]
- False. [ ]

29) The use of birth control pills causes a woman to gain weight and develop acne on her skin.

- True. [ ]
- False. [ ]
